# A new era for *Journal of Experimental & Clinical Cancer Research*

**DOI:** 10.1186/1756-9966-27-1

**Published:** 2008-05-15

**Authors:** Mauro Castelli

**Affiliations:** 1 "Regina Elena" National Cancer Institute, Rome, Italy

*Journal of Experimental & Clinical Cancer Research *(*JECCR*), the official journal of the "Regina Elena" National Cancer Institute [1] of Rome, has moved to BioMed Central's open access publishing platform. Overseen by the Editor-in-Chief Mauro Castelli and supported by an international Editorial Board [2], the journal aims to provide a high-quality forum for basic and translational work in oncology.

First launched in 1982, *JECCR *joined BioMed Central in order to provide rapid dissemination of scientific results to the widest possible global audience. Furthermore, authors submitting to JECCR can expect a fast turnaround time, benefitting from an efficient review process and publication immediately upon acceptance. *JECCR*'s content will be widely indexed (for instance by PubMed, Medline, Thomson/ISI, Embase, and CAS) and covered by a range of freely accessible full-text archives [3].

*JECCR *publishes scientific studies on the biological, epidemiological, immunological, pathological, radiobiological, and clinical aspects of oncology. Topics considered range from molecular genetics via infectious agents to surgery and therapeutic approaches and outcomes. The broad oncological focus, combined with the Editors' experience in evaluating and conducting systematic reviews and developing clinical practice guidelines, makes *JECCR *an attractive forum for publishing reviews and guidelines. This focus, well documented in an Editorial by H. J. Schünemann [4], goes very well together with the approach now being widely applied by many international and national organizations, including the World Health Organization (WHO), Cochrane Collaboration, American College of Physicians (ACP), and the National Institutes for Health and Clinical Excellence in the U.K. (NICE).

In response to increasing requests *JECCR *has decided to devote particular attention to more specific areas of cancer investigation. To this end, specific sections and subsections under the supervision of expert Editors have been created: the new topics include Gastrointestinal Stromal Tumors (GIST), Lung and Pleural Tumors, and Preclinical Investigations in spontaneous animal models.

We believe that the new editorial changes combined with the advantages of BioMed Central's open access publishing platform will greatly improve *JECCR*'s dissemination and visibility. The journal's online archive back to 1999 remains available from its old homepage. [5]
